# Errors in cause-of-death statement on death certificates in intensive care unit of Kathmandu, Nepal

**DOI:** 10.1186/s12913-015-1168-6

**Published:** 2015-11-12

**Authors:** Leison Maharjan, Aarzoo Shah, Khagendra Bahadur Shrestha, Gambhir Shrestha

**Affiliations:** Blue Cross Hospital, Kathmandu, Nepal; Kathmandu Medical College, Kathmandu, Nepal; School of Public Health & Community Medicine, B.P. Koirala Institute of Health Sciences, Dharan, Nepal

**Keywords:** Cause of death, ICU deaths, Errors, Death certificate, Underlying cause of death, Immediate cause of death

## Abstract

**Background:**

Death certificates (DC) are one of the most important medico-legal documents that physicians work through. DCs are extensively used in health statistics for epidemiological studies, and in health policy planning as a public health resource tool. Cause-of-death (COD) statement, which is vulnerable to various errors, is the vital part of a DC that has the potential to mislead the policy makers and statisticians. Hence, we evaluated and analyzed the errors prevalent in COD statement of DC.

**Methods:**

A retrospective observational study was conducted at medical Intensive Care Unit (ICU) of Blue Cross Hospital, Kathmandu, Nepal within two years of study period. A total of 204 medical records of the deceased patients were reviewed. Three sub-headings of COD statement of DC- Part I Immediate COD (ICOD), Part I Underlying COD (UCOD), and Part II Other significant conditions (OSC) were extensively evaluated for the major medical errors.

**Results:**

The study found errors in 78.4 % of DCs. The highest number of errors was in UCOD (83 %). Most common errors were “Mechanism of Death- terminal event” in ICOD, “More than one competing causes” in UCOD, and “OSC present but not listed” in OSC. The error in DC was found to be statistically significant with the severity of sepsis (*p* = 0.003), and presence of chronic organ failures (*p* = 0.034). Age, time of death, source of admission, and duration of ICU stay were not found to be statistically associated with the errors in DC.

**Conclusion:**

Prevalence of errors in DC was quite high. Most errors were committed in underlying cause of death, which is the most important part of DC. Complexity of the cases was the key factor that increased the risks of committing errors. Specific education should supersede general educational interventions to minimize the errors considerably in writing DC in complex cases.

## Background

Death certificate (DC) is a permanent legal record of an individual’s death. It is an important medico-legal document for both the attending physician and relatives of deceased for claiming inheritance and insurances and for recognizing inheritable risk factors [1]. Furthermore, health decision makers and planners all around the world make extensive use of mortality statistics, the quality of which depends on the DCs. DC is also a vital data collection tool for epidemiological studies that contributes to the public health resource pool. Despite its widely acknowledged importance, WHO estimated 2/3^rd^ (38 million out of 56 million) annual deaths globally are still not registered. This is more evident in developing countries as more than half of all deaths occur outside hospitals [2]. Most of the physician-certified deaths come from hospitals since out-of-hospital deaths are rarely medically certified [3, 4]. The physician’s principal responsibility in death registration is to complete the medical part of the death certificate [5]. Although the guidelines on death certification are widely available, they are rarely used in many countries. Therefore, DC is one of the most inaccurately completed documents.

Various studies showed high rate of errors in filling the death certificates. In a study conducted in Atlanta, Hanzlick & Randy MD found about 47 % of the errors in DCs involved omissions, incomplete & incorrect information [6]. Raje MG undertook the study in India where both medical and non-medical errors were evaluated. In the study, it was found that 99 % of DCs were incorrectly written while 21 % were incompletely written [7]. Similar findings were found by Haque et al in a study conducted in Pakistan where 99 % DCs had errors [8]. These errors included minor errors like omission, illegible handwriting, and use of abbreviation and major errors like inaccurate Immediate cause of death (ICOD), Underlying cause of death (UCOD).

Many studies have attempted to find the factors affecting the accuracy of DC statement. A study undertaken in London showed that errors in filling DCs were not significantly better in the cases where autopsies were performed and in the cases where DCs that were signed by coroner instead of house staff [9]. Similarly, a study in Australia claimed that the major error rate was not significantly different among cities and country areas, or between teaching hospitals and other locations [10]. Further, another study showed that the experience of certifying physicians was not associated with improved death certification practice [11]. In this study, we investigated other factors besides certifier’s knowledge and experience which may be significantly associated with the occurrence of error.

Prevalence and types of the errors of DC have been studied widely, but sparse studies have analyzed the various types of error found in different parts of Cause of death (COD) statement. Thus, the aims of our study were to identify the error rate in each part of COD statement of DC and to determine the factors associated with the occurrence of such errors. Patients in ICU have multiple disease conditions and the certifying physicians are more vulnerable to commit errors in presence of complex diagnosis while establishing COD. Thus, we chose to conduct this study in the ICU setting to evaluate the various types of error in DC in detail.

## Methods

### Study setting and design

This is a retrospective observational study undertaken in Blue Cross Hospital, Kathmandu. We reviewed all the death certificates along with patient’s record files from December 2012 to December 2014. Blue Cross Hospital is a tertiary care hospital with a ten-bedded ICU equipped with four ventilators and a dialysis unit. The on-duty Medical Officers filled all the DCs immediately after pronouncing death. The standard format of the DC provided by the Department of Health Services, Government of Nepal was used. DCs from adult deaths (age >14 years) that occurred in ICU were included in the study, while those records where the death occurred within an hour of admission were excluded.

### Analysis of death certificates

We reviewed death certificates to find out the prevalence and types of errors in the COD statement. Only the major errors were evaluated as these errors tend to compromise statistical data, thereby misleading health policy planners and epidemiologists. These errors may also cause hindrance in claiming insurance and confusion during medico-legal procedures.

Non-medical errors such as identification data, administrative data, legible signature/name of certifying physician, and minor COD errors such as use of abbreviations, spelling errors, approximate interval between onset and death, ill-defined conditions followed by adequate UCOD were not evaluated [12, 13].

The COD statement in the DC contains two parts (Table 1). Part I is for reporting the sequence of events leading to death i.e. Immediate cause of death (ICOD), proceeding backwards from the final disease or condition resulting in death, with one condition per line. A specific cause of death i.e. underlying cause of death (UCOD) is reported in the last entry in Part I so that there is no ambiguity about the etiology. Part II is for other significant conditions (OSC) that contributed to the death but did not lead to the underlying cause [5, 14]. COD may vary among individual physicians. In order to consider a properly completed COD section, it must provide an etiologic explanation of the sequence, type, and association of events resulting in death [5]. For each DC to be considered an error-free COD, we used the following criteria in COD statement:

**Table 1:** Cause of death statement of a death certificate with examples

Cause of death	
Part I	
(ICOD)	a) Acute renal failure due to or as consequences of:
	b) Hyperosmolar non-ketotic coma due to or as consequences of:
(UCOD)	c) Diabetic mellitus Type 2
Part II	
(OSC)	Hypertension, Chronic bronchitis

- acceptable ICOD, UCOD & OSC
- proper sequence with no competing causes in UCOD
- absence of any inappropriate information in ICOD, UCOD & OSC
- ill-defined conditions in ICOD, if recorded, should be followed by adequately explained UCOD

We designed a checklist to detect errors in the DC with the help of published guidelines & definitions (Table 2) put forth by International Statistical Classification of Diseases and Related Health Problems (ICD 10 volume 2), World Health Organization (WHO) and *Physicians’ Handbook on Medical Certification of Death* by Centers for Disease Control and Prevention (CDC 2003), National Center for Health & Statistics (NCHS) and Cause-of-Death statements and certification of natural and unnatural deaths by College of American Pathologists (CAP) [5, 15, 16]. The errors in COD statement were further classified under predetermined types (Table 3).

**Table 2:** Operational definitions of some terms derived from International Statistical Classification of Diseases and Related Health Problems (ICD-10) and College of American Pathologists (CAP) [15, 16]

Causes of death: “all those disease, morbid conditions or injuries which either resulted in or contributed to death and the circumstances of the accident or violence which produced any such injuries”. (Twentieth World Health Assembly, 1967)	
Immediate cause of death: The final disease or injury causing the death.	
Underlying cause of death: “(a) the disease or injury which initiated the train of morbid events leading directly to death, or (b) the circumstances of the accident or violence which produced the fatal injury”. (World Health Organization, 1994)	
Part II is for any other significant condition that contributed to the fatal outcome, but was not related to the disease or condition directly causing death.	
“due to (or consequences of)”: after these word on the certificate, should be included not only the direct cause or pathological process, but also indirect causes, for example where an antecedent condition has predisposed to the direct cause by damage to tissues or impairment of function, even after a long interval.	
Sequence: refers to two or more conditions entered on successive lines of Part I, each condition being an acceptable cause of the one entered on the line above it. If there is more than one cause of death in a line of the certificate, it is possible to have more than one reported sequence.	
Ill-Defined conditions: I46.9 (Cardiac arrest, unspecified); I95.9 (Hypotension; unspecified); I99 (Other and unspecified disorders of circulatory system); J96.O (Acute respiratory failure); J96.9 (Respiratory failure, unspecified); R00-R94 and R96- R99 (Symptoms, signs and abnormal clinical and laboratory findings, not elsewhere classified).

**Table 3:** Classification of types of error in cause of death statement of death certificate

Parts of cause of death statement	Types of errors	Examples
Part I Immediate Cause of Death (ICOD)	Not listed	
	Inappropriate information	Incorrectly attributed/Trivial conditions
	^a^Mechanism of Death *(terminal events)*	Such as asystole, cardiac arrest, cardiopulmonary arrest, cardiorespiratory arrest, electromechanical dissociation, respiratory arrest, ventricular fibrillation.
	^a^Mechanism of Death *(nonspecific physiologic derangements*)	Such as arrhythmia, coagulopathy, congestive heart failure, hepatic encephalopathy, hepatic failure, hypotension, ketoacidosis, multi-organ failure, pneumothorax, pulmonary insufficiency, renal failure, sepsis, shock.
	^a^Nonspecific anatomic processes	Such as anoxic encephalopathy, bowel obstruction, cirrhosis, gastrointestinal hemorrhage, hemothorax, peritonitis, pulmonary embolism, subarachnoid hemorrhage, subdural hematoma.
	^a^Symptoms, signs, abnormal clinical and laboratory findings	Such as headache, chest pain, dyspnea, asterixis, pain abdomen, hyperkalemia, hypercalcemia
Part I Underlying Cause of Death (UCOD)	^b^Not listed	
	Inappropriate information	Incorrectly attributed/Trivial conditions
	Incomplete information	Ill-defined conditions, unspecific and incomplete description of known specific conditions such as neoplasms, infectious diseases, injuries, external causes of death
	More than 1 competing UCOD	2 or more unrelated conditions listed
	Improper sequence	sequence of events doesn’t make sense, UCOD not listed on the lowest completed line of Part I
Part II Other Significant Conditions (OSC)	Not listed	OSC present but not listed
	Inappropriate information	Incorrectly attributed/Trivial conditions
	Incomplete information	not all the known significant conditions are listed

^a^Ill-defined conditions not followed by adequately explained UCOD

^b^ICOD can be the sole entry in COD statement if that condition is the only condition causing death. In such a condition, it is not considered as an error if UCOD is not listed

The initial analysis of the cause of death (ICOD, UCOD, OSC) was done independent of patient’s DC from the information available in patients’ medical records. It contained detailed admission records, hospital stay summary, nurses’ progress note, clinical progress note and laboratory investigations. Thereafter, we cross examined the accuracy of the DC information. Each case was broadly categorized into three categories of Sepsis - a) ‘No sepsis’ that included cases with no Infection/Systemic Inflammatory Response Syndrome (SIRS); b) ‘Infection/SIRS/Sepsis’ that included SIRS, Infection, Sepsis, Severe sepsis and Septic shock; and c) ‘Multiple Organ Dysfunction Syndrome (MODS)’ that included cases with two or more organ dysfunctions with each organ dysfunction defined by MSOFA (Modified Sequential Organ Failure Assessment) score more than or equal to 1 [17, 18]. No autopsy was performed in any of the cases. There were no pregnancy related cases within the study period. The causes of death in DCs were classified according to ICD – 10.

Two investigators independently evaluated the DCs and in case of discrepancy, the opinion of the third investigator was taken and was considered final.

### Statistical analysis

All the data were entered in MS-EXCEL 2007 software and analyzed in Statistical Package for Social Sciences (SPSS, version 17). Dependent variable was the occurrence of error in DC while independent variables were gender, age, time of deaths, duration of ICU stay, and presence of infection (sepsis), MODS and chronic organ failures.

Descriptive data analysis was used to show the prevalence and types of errors in DC and the other related variables. Comparative study was done amongst different types of errors prevalent in ICOD, UCOD and OSC. Chi-square test was used to determine the association of occurrence of error in DC with the independent variables. A *p*-value of <0.05 was considered as the cut-off point for statistical significance.

### Ethical consideration

The study was reviewed and approved by Nepal Health Research Council. Approval was also obtained from the Hospital Director of Blue Cross Hospital to use the records of the patients.

## Results

A total of 220 deaths were recorded out of 1055 admissions in the ICU of Blue Cross Hospital during the two years study period. Of which, 13 record files were not accessible and three cases were excluded from the study as death occurred within an hour of ICU admission. Hence, 204 record files were evaluated in the study. Among the deaths, 60.8 % were male and 39.2 % were female with mean age of 57.89 years (SD = 19.23). The mean duration of ICU stay was 3.38 days (SD = 4.65). About 41.2 % deceased had chronic organ failure, and majority of them (80.9 %) had multiple organ dysfunction syndrome. Respiratory diseases accounted for 30.2 % of deaths in this study (Table 4).

**Table 4:** Characteristics of deceased in ICU of Blue Cross Hospital (*N* = 204)

Characteristics	Number (n)	Percentage (%)
Age in years (mean ± SD)	57.89 ± 19.23	
Gender		
Male	124	60.8
Female	80	39.2
ICU stay in days (mean ± SD)	3.38 ± 4.65	
Chronic organ failure		
Absent	120	58.8
Present	84	41.2
Sepsis classification		
No sepsis	31	15.2
Infection/SIRS/Sepsis	8	3.9
MODS	165	80.9
Causes of death According to ICD – 10		
Diseases of the respiratory system	62	30.4
Diseases of the nervous system	34	16.7
Diseases of digestive system	33	16.2
Diseases of the genitourinary system	33	16.2
Neoplasms	14	6.9
External causes of morbidity and mortality	12	5.9
Diseases of the circulatory system	6	2.9
Poisoning	5	2.5
Others	5	2.5

**Fig. 1:**
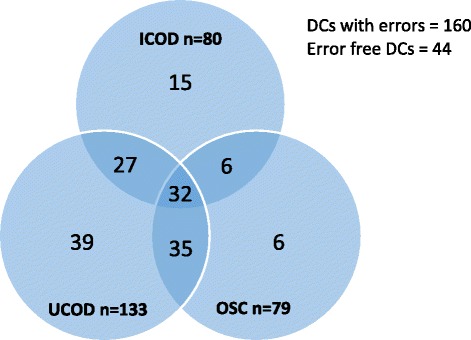
Venn diagram showing distribution of errors in different parts of death certificates

In this study, we found errors in 160 (78.4 %) DCs. The highest error rate was found in sub-heading UCOD in 133 DCs followed by ICOD in 80 DCs and OSC in 79 DCs. About 20 % of 160 erroneous DCs had errors in all three subheadings (ICOD, UCOD and OSC), whereas 68 DCs and 60 DCs had errors in two and one subheading respectively (Fig. 1).

Altogether, there were 334 errors in the DCs, comprising of 29.0 % in ICOD, 46.4 % in UCOD and 24.6 % in OSC parts. The most common error were “Mechanism of Death- terminal event” in ICOD, “More than one competing causes” in UCOD, and “OSC present but not listed” in OSC (Table 5). Though certifiers mostly committed single error in a sub-heading (*n* = 251), two errors (*n* = 40) and three errors (*n* = 1) were also committed within the same sub-heading. Among two errors committed within the same sub-heading, combination of “more than 1 competing causes” and “inappropriate information” in UCOD was found to be the highest (20 DCs) (Table 6).

**Table 5:** Frequency of errors in different parts of death certificates (*N* = 204)

Parts of cause of death statement	Types of errors	Number (n)	Percentage (%)
Part I Immediate Cause of Death (ICOD)	Not listed	0	0.0
	Inappropriate information	8	2.4
	Mechanism of Death *(terminal events)*	58	17.4
	Mechanism of Death *(nonspecific physiologic derangements*)	28	8.4
	Nonspecific anatomic processes	2	0.6
	Symptoms, signs, abnormal clinical and laboratory findings	1	0.3
	Total errors in ICOD	97	29.0
Part I Underlying Cause of Death (UCOD)	Not listed	12	3.6
	Inappropriate information	33	9.9
	Incomplete information	30	8.9
	More than 1 competing UCOD	49	14.7
	Improper sequence	31	9.3
	Total errors in UCOD	155	46.4
Part II Other Significant Conditions (OSC)	Not listed	47	14.1
	Inappropriate information	33	9.9
	Incomplete information	2	0.6
	Total errors in OSC	82	24.6
Total errors in death certificates		334*	100.0

*A death certificate may contain more than 1 error

**Table 6:** Distribution of numbers of errors in death certificates committed within single subheading (*N* = 204)

No. of errors committed within single subheading	ICOD (n)	UCOD (n)	OSC (n)	Total (n)
One	63	112	76	251
Two	17	20	3	40
Three	0	1	0	1
Total	80	133	79	-

Our study also found that the occurrence of errors was significantly associated with the severity of sepsis (*p* = 0.003) and the presence of chronic organ failure (*p* = 0.034). However, association with other factors such as gender, age, duration of ICU stay and time of death were not statistically significant (Table 7). The highest percentage of errors was found in deaths due to external causes of morbidities and mortalities (91.7 %), while the lowest percentage of errors was found in deaths due to poisoning (60.0 %) & diseases of the nervous system (61.8 %) (Table 8).

**Table 7:** Association of errors in death certificates with various factors (*N* = 204)

Variables	Errors in death certificate		*P*-value
	Absent n (%)	Present n (%)	
Gender			
Male	25 (20.2 %)	99 (79.8 %)	0.543
Female	19 (23.8 %)	61 (76.2 %)	
Age in years			
14 – 30	8 (33.3 %)	16 (66.7 %)	0.122
30 – 45	11(34.4 %)	21 (65.6 %)	
45 – 60	7 (14.3 %)	42 (85.7 %)	
60 – 75	11 (18 %)	50 (82 %)	
>75	7 (18.4 %)	31 (81.6 %)	
ICU stay in days			
< 3	33 (22.4 %)	114 (77.6 %)	0.779
3 – 6	6 (22.2 %)	21 (77.8 %)	
> 6	5 (16.7 %)	25 (83.3 %)	
Sepsis classification			
No sepsis	7 (50.0 %)	7 (50.0 %)	0.003*
Infection/SIRS/Sepsis	9 (36.0 %)	16 (64.0 %)	
MODS	28 (17.0 %)	137 (83.0 %)	
Chronic Organ Failure			
Absent	32 (26.7 %)	88 (73.3 %)	0.034*
Present	12 (14.3 %)	72 (85.7 %)	
Time of death			
Day – evening	34 (25.6 %)	99 (74.4 %)	0.058
Night time	10 (14.1 %)	61 (85.9 %)	

*Statistically significant at p < 0.05

**Table 8:** Distribution of errors in death certificates with causes of death according to ICD-10 classification (*N* = 204)

Causes of death	Errors in death certificate	
	Absent n (%)	Present n (%)
Diseases of the respiratory system	14 (22.6 %)	48 (77.4 %)
Diseases of the nervous system	13 (38.2 %)	21 (61.8 %)
Diseases of the digestive system	5 (15.2 %)	28 (84.9 %)
Diseases of the genitourinary system	4 (12.1 %)	29 (87.9 %)
Neoplasms	4 (28.6 %)	10 (71.4 %)
External causes of morbidity and mortality	1 (8.3 %)	11 (91.7 %)
Diseases of the circulatory system	1 (16.7 %)	5 (83.3 %)
Poisoning	2 (40 %)	3 (60 %)
Others	0 (0 %)	5 (100 %)

## Discussion

Death certificate is a vital statistical tool for health policy planners, public health researchers, and epidemiologists. A complete and accurate death certificate is necessary in order to reflect and gain actual data. The absence of reliable data on causes of death impedes the structuring of health-related activities and results in misleading appraisals of research and improper decisions regarding health care policies [8]. Medical students and interns all over the world are taught about DC [19]. Despite this, the error rate is significantly high globally.

In this study, we found 78.4 % DCs erroneous. The rate of major error was significantly higher in this study as compared to other studies. Pritt et al found 34 % DC with major error [20]. In various other studies, the major error rate ranged from 24 to 37 % [9, 13, 19, 21]. A plausible reason for such a significant difference in the result may be due to the variation in the criteria used to define major errors. Another reason could be that our study is limited to DCs of the medical ICU only. The mortality rate is higher in medical ICU and the chances of committing major errors are maximum due to the presence of multiple co-morbid conditions in the ICU patients.

Our study found the highest error rate in UCOD (46 %). One of the reasons could be the multiple co-morbid conditions present in a single patient that makes it difficult for the certifier to sort out the actual disease that led to the death of a patient. In hospitals, it is mainly the on-duty doctors who certify the DC, and there is a high chance that the certifying doctor may not have examined the patient previously. Therefore, lack of detail prior knowledge of the case may prompt the certifier to commit these errors. Another reason could be that the hectic duty hours in the ICU limit the time to review the medical files of the deceased thoroughly. This causes the certifier to miss the vital information required to fill the UCOD part of DC. UCOD mainly reflects the disease that led to the death of the patient. Hence, an error in UCOD will significantly misinform and compromise the data leading to misguidance on the actual burden of disease prevalent in the society.

The most common errors in individual sub-heading were “Mechanism of Death- terminal event” in ICOD, “More than one competing causes” in UCOD, and “OSC present but not listed” in OSC. It might be due to the lack of knowledge of completing DC or decreased perceived importance of DC. A study found that 46.2 % of the House Officers had not read the instructions book on death certificate [11]. Furthermore, physicians often do not receive adequate training in standard ICD death certification practices. It is, therefore, not surprising that comparative assessments commonly find that the quality of medical certification of the cause of death is poor [4, 22]. Among the “not listed” errors in different parts of DCs, OSC had the highest number of errors (*n* = 47) followed by 12 errors in UCOD and no error in ICOD. This indicates that certifiers gave less importance to filling Part II of DC or OSC. “Inappropriate information” was high in UCOD and OSC parts. This may be due to the habit of listing the comorbid conditions especially in OSC irrespective of whether the condition led to death or not. This might suggest a lack of confidence in clinical judgment or a lack of knowledge of co-morbid conditions of the patient. The study conducted by James and Bull showed that inaccuracies in DCs arose from inadequate formulation of cause of death and failure to record relevant information [23]. The second most common error in ICOD part was “mechanism of death-nonspecific physiology”. The reason may be due to the fact that most of the treatments are directed towards correcting physiological parameters, hence confusing the certifier. Similarly, we also found that the high rate of errors was in UCOD under “improper sequence” which may be due to the inability of certifier to arrange the clinical events in proper sequence. It is immensely important to address this error properly because the same sequence of events if interchanged leads to different coding of UCOD.

In our study, we found single error (*n* = 251) often committed under individual sub-heading than two errors (*n* = 40) and three errors (*n* = 1). Amongst two errors, frequent errors were committed in UCOD. “More than 1 competing cause” was frequently accompanied by either “Inappropriate information (Incorrectly attributed/ trivial conditions)” or “Improper sequence” under UCOD sub-heading. It might indicate the attempt made by the certifier to find out the actual underlying cause. Enlisting more than one cause reflects the sincere effort of the certifier of not missing the actual cause of death in presence of complex working diagnosis. This is in accordance with the study done by Fernando R. where the immediate, antecedent, and contributory causes of death were not found to be written in the correct place in 30 % certificates, while unacceptable causes of death were found in 19 % [24].

In our study, the least number of errors was found in poisoning (60.0 %) & diseases of nervous system (61.8 %). This could be because of the diagnoses in such cases are quite straight forward, making it easy for the certifier to fill up the COD statement. Poisoning agents can be easily detected e.g. Dichlorovous poisoning, Methanol poisoning. Similarly, diagnostic imaging and other facilities can accurately diagnose neurological diseases e.g. Right anterior communicating artery aneurysm rupture, Japanese encephalitis etc., and thus eliminates the certifier’s dilemma. “Incomplete information” under UCOD was found more often in external causes of morbidities and mortalities, neoplasms, and diseases of digestive system. The possible reason for this finding could be the habit of the certifier of writing an incomplete diagnosis. Not mentioning about the mechanism of accidents in trauma cases (e.g. Road traffic accident instead of ‘hit by a bike’) results in missing out vital information in UCOD. Likewise failing to mention the pathological diagnosis and grade in cases of malignancies (e.g. Adenocarcinoma lung instead of ‘well differentiated Adenocarcinoma grade I in Right lobe of Right lung with hilar lymphnodes metastasis’) and the specific anatomical site (e.g. Upper gastrointestinal bleeding instead of ‘peptic ulcer bleeding’) in diseases of digestive system can be labeled as an incomplete information. The study conducted by James and Bull showed that the histological diagnosis was available in 79.1 % of cases of deaths due to malignancy but was recorded on only 23.6 % of certificates which is in concordance with our finding [25].

We also found that the rate of errors in DCs significantly increased with the presence of chronic organ failure. The possible reason for this may be due to the presence of multiple dysfunction and biochemical derangement in patients with chronic organ failure. This further confuses the certifier to identify the actual cause of death. Organ failure does not occur as an all or none rule. Instead, a range of organ dysfunction exists resulting in the clinical organ failure [26]. Moreover, failure of one organ leads to failure of other organs over course of time thus adding more dilemmas while filling the COD statement. We also found statistically significant association between the error rates and the severity of sepsis. As severity increases, the number of organ dysfunction and failure also increases; thus complicating the whole disease process and further confusing the certifier with multiple options and inciting them to commit errors. We found that the error rate increased in presence of MODS. Similarly, we found increase in the error rate as the duration of ICU stay increased. The probable explanation may be because of the fact that the disease process likely deteriorates and progresses to multiple consequences with time in ICU patients. Thus complicating the cases and increasing the difficulty in writing the COD. Likewise, more errors were seen in DC written during the night, which could be a result of fatigue [27].

There are some limitations in our study. It cannot be generalized as our study is retrospective and was carried out in an adult medical ICU of a centrally located tertiary hospital. We evaluated only the major medical errors in the DC. The sample size was also small. The error rates would have been even higher had we included the minor errors such as omission, use of abbreviations or non-medical part of DC. We also did not assess the knowledge and experience of certifying medical officers. Due to socio-cultural reasons, autopsy was not performed in any of the cases.

We recommend DC completing skills be taught not only to medical undergraduate students but also revised to interns, medical officers, and residents. DC completing skills should also be a part of continuing medical education. Studies have shown such errors can be minimized with the implementation of a simple educational intervention [13, 28]. Moreover, to carry out a validation study of cause-of-death data collection in hospitals, a gold standard framework is required, against which the hospital cause-of-death reports can be compared [29]. This further assures the accuracy of DCs.

Besides, in developing countries where most of the deaths go uncounted, measures to register such deaths as per WHO recommended verbal autopsy questionnaires must be encouraged. This will help to improve the accuracy of causes of death from systems without medical certification of death [2]. Hence ensuring the actual cause of death data collection in the resource pool and increasing the quality of the data.

## Conclusions

The prevalence of errors in the DCs in our study was found to be quite high. Most errors were committed in underlying cause of death section; the most important part of DCs. Complexity of the cases was the key factor that increased the risks of committing errors. Rather than general educational interventions, specific education targeted towards writing DCs in complex cases to considerably minimize the errors should be formulated. Furthermore, a gold standard framework to compare and analyze the accuracy of DCs in the hospitals must be devised.

## Abbreviations

DC: Death certificate
ICU: Intensive Care Unit
COD: Cause of death
ICOD: Immediate cause of death
UCOD: Underlying cause of death
OSC: Other significant causes
ICD: International Statistical Classification of Diseases and Related Health Problems
WHO: World Health Organization
CDC: Centers for Disease Control and Prevention
NCHS: National Center for health & statistics
CAP: College of American Pathologist
MODS: Multiple organ dysfunction system
MSOFA: Modified sequential organ failure assessment score
SPSS: Statistical package for social science
SD: Standard deviation
